# Geroscience and the challenges of aging societies

**DOI:** 10.1002/agm2.12082

**Published:** 2019-10-10

**Authors:** Felipe Sierra

**Affiliations:** ^1^ Division of Aging Biology National Institute on Aging (NIA) National Institutes of Health (NIH) Bethesda MD USA

The world is aging. With both an increase in longevity and a decrease in fecundity, currently there are more individuals over 65 years of age than children aged <5 years. This is fully uncharted territory for humanity. The process is worldwide. While the process has been slow in more developed societies, it has been extremely rapid in developing ones, causing severe turmoil. In addition to the personal cost in terms of a large and growing number of individuals suffering from diseases, loneliness, neglect, and lack of personal support, aging of the population implies increased costs in health care, as well as a dramatic need for increased resources needed to sustain a growing nonproductive fraction of society. As a result, the traditional view is that this demographic change represents an enormous social, personal, and economic challenge to modern society.

But is this so? Every cloud has a silver lining, and perhaps the aging of the population represents an opportunity instead. After all, population aging is the result of outstanding advances in medicine and public health that have led to improvement of sanitation and conquering of many diseases and conditions. Success in the early 20th century was primarily in the containment of early childhood mortality, childbirth mortality for women, and virtual eradication of many transmissible diseases, thanks to actions on public health and hygiene as well as the development of vaccines. More recently, trends in extended longevity have also been observed for the >50‐year‐old population, implicating successes in the fight against major killers, such as cardiovascular disease, cancer, and others. As a result, mortality rates have decreased for all age groups, including the oldest‐old. This reflects reduced morbidity at all ages and it means that a 75‐year‐old “elderly” person now has a physiological age and vitality comparable to that of a 65‐year‐old of just one to two decades ago. It should be possible to continue progressing with this enormous success, and viewed in this context, the so‐called aging tsunami represents instead an opportunity: the longer individuals keep their health, the higher their productive potential during their lifetime. Indeed, studies have shown that retarding frailty by just a couple of years yields an economic dividend of billions of dollars on health‐care savings, even without considering the potential for continued productivity.^1^ Therefore, if properly handled, aging of the society actually represents an economic and social opportunity, not a burden or a source of concern.^2^ However, to take full advantage of this opportunity, it will be necessary to modify our approach to combating the chronic diseases that are the major blockage to achieving productive and healthy lives as we grow older. The current predominant approach in biomedicine, at least in Western societies, is to attack diseases one at a time and with a strong emphasis on those diseases with the highest mortality, at the expense of other conditions, such as chronic pain, frailty, and fatigability, which—without being deadly—truly rob older adults of their quality of life. This model has some advantages, as people afflicted by those diseases are seeing an extension of their life spans and their diseases being, if not cured, at least managed. However, at the population level, it is estimated that completely curing cancer and cardiovascular disease will only add a few years to the overall life span.^3^ This is because, unfortunately, older persons rarely suffer from just one disease, a situation termed “multimorbidity” or “comorbidity.” This means that postponing and/or treating one deadly disease often means that the individual will be affected soon after by another one, so the gain in longevity is not as large as anticipated. Work by Crimmins et al^4^ has shown that the increased longevity achieved in the last few years and decades has been accompanied by an even larger increase in the incidence of chronic diseases that affect primarily older adults. Indeed, while both longevity and disease‐free survival have seen significant improvements in the last century, their rates differ, so that the rate of increase in morbidity surpasses the rate of increase in longevity, leading to longer but unhealthy lives. As a result, society as a whole experiences increased expenses in health care, and as individuals we live our senior years without a true improvement in our quality of life.

What is the solution? Where is that silver lining? It is not by mere chance that most chronic diseases and disabilities appear with advancing age independently of the genetic or environmental endowment of the individual. The reason is simple: while not a disease itself, the biological process of aging is by far the greatest risk factor for all chronic diseases. There are many misconceptions about aging, and many confuse chronological and physiological age. Our chronological age cannot be manipulated, but we know that physiological fitness in old age can be affected by simple and well‐known factors, such as exercise, diet, and cessation of smoking. And we know that such actions can modify the appearance of chronic diseases and conditions. Thus, it is not “age” (the passing of time) but “aging” (the process) that can be manipulated. The passing of years allows for the accumulation of molecular damage, which many equate with aging, the process. However, while the young organism is endowed with enough resilience to remove most of this damage, during the process of aging our organism partially loses its capacity to cope with further damage, leading to the appearance of diseases and disabilities. It is not simply that “the accumulated damage overwhelms the defense mechanisms,” as many scientists and physicians believe. It is instead that we lose the capacity to defend against that damage. Take the example of Alzheimer's disease: it is rare to see amyloid deposits in people before age 40 years,^5^ and even in individuals with aggressive mutations in Amyloid Precursor Protein (APP) or presenilins, no plaques are observed in childhood or early adulthood, in spite of massive amounts of misfolded proteins being produced. This is because youthful resilience resolves much of the damage by elimination of toxic byproducts, possibly by segregating them into less toxic aggregates. Of course, no process is 100% efficient, so some accumulation does indeed occur throughout life, but it is only after the defense capacities decline that this lifelong minor accumulation, coupled with ongoing further damage, overwhelms the defense system, and the disease appears.

The fact that aging is the major risk factor for chronic diseases in older adults, while intuitive, has not been adequately exploited in medicine because aging is often seen as “inevitable” and equated with chronological age. An additional roadblock has been the perceived utter complexity of the process, which has led to inadequate efforts at acquiring scientific knowledge and understanding about the basic molecular and cellular mechanisms that control the aging process. However, this last issue has changed dramatically during the last two decades, and scientists now have a good grasp of a half‐dozen “pillars of aging” (such as inflammation, stress response, and epigenetics) that drive the physical symptoms and appearance of aging frailties.^6^, ^7^ This in turn has allowed the recent development of promising pharmacological therapies. The first breakthrough was the identification by the Interventions Testing Program of the NIA in 2009 that rapamycin extends the life span in four‐way cross mice.^8^ The result has been reproduced in many other strains of mice as well as multiple other species. Most importantly, however, multiple studies have shown that rapamycin extends longevity by providing protection against multiple diseases and other challenges, thus showing that, in addition to life span, rapamycin increases health span. Of course, like any other drug, there are also some negative effects, most notably on glucose handling in mice. There is also a concern in the medical profession that rapamycin (and rapalogs) have been associated with multiple negative side‐effects. However, data from a recent study in humans have shown a dramatic improvement in vaccine responsiveness without significant side‐effects.^9^ Similar studies are being conducted in pre‐clinical settings, using pet dogs and marmosets. Many other drugs that extend the life spans (and sometimes the health spans) of mice and other species by addressing aging instead of specific diseases have been described in recent years, but the lion's share of recent interest has been on senolytics and NAD^+^ precursors. The data on the latter are minimal and not well documented, though the little that is known appears very promising. The story on senolytics is more compelling, as the field of cellular senescence has been controversial since its outset in the early 1960s, but two relatively recent developments brought the field to the fore: the discovery of a largely “negative” secretory phenotype (Senescence‐Associated Secretory Phenotype [SASP]), and the demonstration, by genetic means, that selective removal of senescent cells leads to dramatic improvements in both life span and health span. Many drugs that selectively (though not uniquely) kill senescent cells have been identified by multiple approaches, and senolytics have recently been used in clinical studies in humans.^10^ While preliminary and underpowered, the results are encouraging. An important point to emphasize is that these pharmacological approaches are aimed at the core process of aging, rather than the disease or condition under study directly. For example, it is interesting to note that rapamycin, a well‐known immune suppressor, had a positive effect precisely on the immunological response to vaccines.

In addition to pharmacological means of extending the life span and health span, several novel dietary paradigms are currently being tested in multiple laboratories. These variations on the old diet‐restriction paradigm of aging research focus primarily on the effect of circadian control of diet (ie, when we eat, not what or how much we eat), as well as new knowledge about the metabolic value of intermittent fasting,^11^ with or without limiting calories. These are exciting new avenues of research that still require much further study.

Within this context came the field of geroscience, which aims to understand at the molecular and cellular level the mechanisms by which aging leads to an increase in the risk for multiple chronic conditions^12^ (see Figure 1). Geroscience acknowledges the simple hypothesis that an individual's genes and environment will determine which diseases and disabilities they will suffer from as they age, but the process of aging (ie, the loss of resilience) will determine when these events become evident. If we accept that the process of aging is the major driver of all chronic ailments affecting the aged population, then it becomes clear that addressing one disease at a time is not a winning strategy within the context of multimorbidity, and it should be more effective to address aging instead, as a way to stave off the entire array of disorders. Thus, the hypothesis behind this concept is that if we attack aging directly, we will be able to prevent or delay multiple chronic diseases and other ailments, thus improving the quality of life of the “elderly.”^13^ Multiple lines of evidence in animal models support this concept, and emerging evidence from small clinical trials has begun to test this hypothesis in humans. The goal is to evaluate interventions that could delay weaknesses and diseases that characterize old age, pushing back the onset of frailty. This would reduce the fraction of the life span in which people experience the discomforts of old age. An important side‐effect is that, as a result, health‐care expenditures would be reduced both by the individual and by society. This would be a seismic change in the way we approach medicine, and it would allow health‐care expenditures to be focused on the smaller proportion of the population that, because of their genes and/or environment, inevitably will not be able to profit from the new approaches.

**Figure 1:**
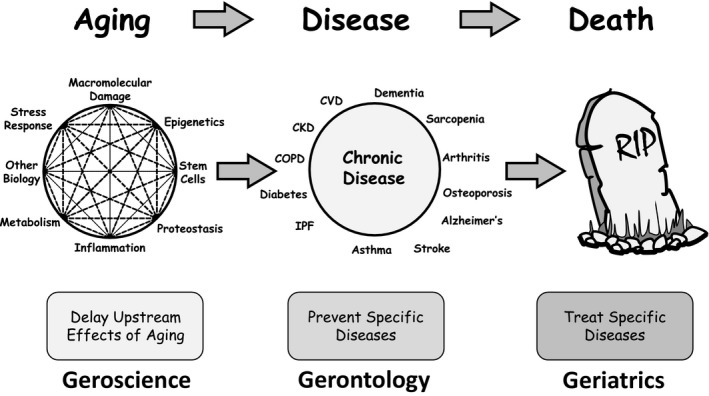
Geroscience and related disciplines. The *Webster‐Merriam Dictionary* defines “geriatrics” as: “A branch of medicine that deals with the problems and diseases of old age and the medical care and treatment of aging people.” It further defines “gerontology” as: “The comprehensive multidisciplinary study of aging and older adults.” In contrast, geroscience is a new field (not yet in the *Webster‐Merriam Dictionary*) best defined as a multidisciplinary approach that seeks to understand the genetic, molecular, and cellular mechanisms that make aging a major risk factor and driver of common chronic conditions and diseases of older people. The geroscience hypothesis posits that since aging physiology plays a role in many—if not all—chronic diseases, therapeutically addressing aging physiology directly will prevent the onset or mitigate the severity of multiple chronic diseases. CKD, chronic kidney disease; COPD, chronic obstructive pulmonary disease; CVD, cardiovascular disease; IPF, idiopathic pulmonary fibrosis

In addition to these savings in health care, the expected increase in health span will afford the expansion of productive years, thus bringing along important additional economic dividends. As a note of caution, simply increasing the age of retirement is not palatable to many in society, and it is politically fraught. Therefore, new approaches and policies will be necessary, including re‐education after retirement and in many ways, large and small, making economic, social, and affective space for the so‐called fourth age. This will require vigorous education of the younger members of the society to combat the negative effects of rampant “ageism,” the view that older adults are a useless burden, belonging to a stage that does not participate or produce. Thus, communicating a new outlook might be considered as one more significant element in the development of a prosperous and healthy future world.

## CONFLICTS OF INTEREST

The author discloses no conflicts of interest. The ideas discussed represent Dr. Sierra's views and do not represent the views of the US government.
